# The Many Lives of Darwin’s Letters

**DOI:** 10.1007/s10739-022-09685-6

**Published:** 2022-06-24

**Authors:** Paul White

**Affiliations:** Darwin Correspondence Project, University of Cambridge, Cambridge, UK

**Keywords:** Darwin, Letters, Biography, Character, Public, Private

## Abstract

The *Correspondence of Charles Darwin* will be completed in 2022. This essay looks briefly at the history of editing Darwin and compares the modern edition with the Victorian practice of narrating an exemplary life through letters, with commemorative volumes produced by a family member or friend and private material carefully selected to document personal character. How is a scientific life composed? What kind of character is expressed in scientific work?

In 1974, Frederick Burkhardt was about to embark on an edition of Thomas Huxley’s letters when he was reminded that Darwin’s correspondence had yet to be published. Would it not be better to do that first? Of course, Darwin had received a handsome tribute in the *Life and Letters* edited by his son Francis: three volumes in 1887 and two more thereafter. But Francis Darwin had followed the practices of the period, selecting letters, excising portions, removing names and any content deemed too private or likely to offend. Transcriptions were not always reliable, and dates were sometimes missing or inaccurate. Burkhardt and his co-editors set out to produce an “authoritative” and “definitive” work. What did that mean? At the time, there were few precedents for publishing scientific correspondence. It was, however, the dawn of the Darwin industry. The letters would receive the same painstaking attention as Darwin’s marginalia, notebooks, and other manuscripts on species. The principles of meticulous textual scholarship were laid out in a preface to the first volume (Burkhardt et al. eds. 1985–2022, vol. 1, pp. xxv-xxix). Darwin’s often craggy handwriting became sacred, and every punctuation mark, pen rest, and deletion became a guide to his thought. Much has been gained by this modern edition. It is a rich resource for Darwin specialists, historians of evolutionary theory, Victorianists, scholars, and students of science communication and network theory, to name a few. But it is also worth asking what has been lost in the process, when the veracity of the letter is paramount and the individual *life* is broken into thousands of fragments with no guiding narrative or connecting thread?

As a Victorianist, I often consulted the *Lives and Letters* of Huxley, Hooker, and many others. I became interested in how the genre emerged as a form of biography. The editorial practice of dutiful selection, correction, and omission is easily dismissed as a byproduct of Victorian respectability. But it has a more interesting history: it occupies a curious place in the development of letter writing and publishing. Editors of Victorian *Lives* embraced their role as guardians of “the inner sanctuary of the heart” and the “sanctities of domestic life” (Bunsen 1852, p. vii; Young 1871, p. xi). The genre helped draw new public and private boundaries and police this borderline in science. It also played a significant role in defining models of individuality based on *character*, including the scientific self. “In choosing letters for publication,” Francis wrote, “I have been largely guided by the wish to illustrate my father’s personal character” (F. Darwin ed. 1887, vol. 1, p. iii). How was a scientific life composed? What kind of character was expressed in scientific work?

Throughout the early modern period, letters were often read aloud and circulated to third parties. Collections were compiled for wit, wisdom, and repartee. Around the turn of the nineteenth century, letters grew increasingly “private” (Chartier 1997; Dirks 1999). They became expressions of inner character, a moral quality beneath the surface of rank and title, outward appearance, manners, and even language (Collini 1991, pp. 91–118; White 2006). Character was often difficult to assess. The problem was exploited to dramatic effect in Jane Austen’s novels, where crucial details of personal history were withheld, and ignorance of true character had dire consequences. Anne Secord’s work on correspondence has shown how letters could make character legible, establishing trustworthiness between persons of different social station (Secord 1994). The *Life and Letters* emerged in the early nineteenth century just as the epistolary novel began to decline as a literary form. “Real” letters came to represent a new type of personal character that was developmental, cumulative, and marked by interiority. Correspondence is “always of interest,” wrote Charles Knight, when “it is not a mere effort of authorship, but unfolds the real character of the individual who puts himself before us in an undress” (Knight 1867, p. v). Thus, letters written without regard for publication became the primary material for documenting a life in print.

The new genre developed slowly, with only a handful of titles a year. At first, it embraced poets, churchmen, and statesmen, but it became a standard biographical form by the middle decades. The *Life and Letters* enabled a shift from heroic accounts centered on “great works” to exemplary lives in which virtue was displayed in daily routines, disciplines, and feelings. Even the lives of famous historical figures were recast as homely, epistolic histories: St. Paul, Cicero, and Cromwell, their “inward lives … springs of action … minute developments, and peculiar individual circumstances” revealed to modern readers for edification and emulation (Carlyle 1845; Jeans 1839; Conybeare and Howson 1850, pp. vii-xi). “Men of science,” a learned profession that began to form in the 1830 and 1840s, gradually joined the field. First came Humphrey Davy (Davy 1836), then Isaac Newton (Brewster 1855), Edward Forbes (Wilson and Geikie 1861), and Michael Faraday (Jones 1870). Whatever the details, a scientific life was one of tireless devotion to truth, indifference to personal gain, and the absence of worldly ambition. Darwin’s *Life* was unusual in including a lengthy “autobiography” and reminiscences from his son. Darwin remarked on his passion for science and emphasized the unremitting labor that had produced *Origin of Species* and the many books that followed. His self-portrait was dry, however, and Francis, who had worked closely with his father during the last six years of his life, added color to Darwin’s sketch, describing his “love for each particular experiment,” his “eager zeal not to lose the fruit of it,” and the excitement he took in the most detailed observations, counting seeds under a microscope with keen alertness: “I think he personified each seed as a small demon trying to elude him by getting into the wrong heap … he had an affection, half-artistic, half-botanical, for the little blue Lobelia” (F. Darwin ed. 1887, vol. 1, pp. 116–117, 144–147).

With rare exceptions, women’s lives were not commemorated in such volumes, and their work, scientific or otherwise, was unacknowledged. They were assigned a supportive role as wives and mothers, nurturing the home in a world of struggle. However, women often functioned as editors, determining what material would surface and what would be suppressed. Sometimes this was explicit. Charles Lyell’s *Life* was edited by his sister-in-law Katharine; Charles Kingsley’s by his wife Fanny. In other cases, women worked less conspicuously. Leonard Huxley’s *Life and Letters of Joseph Hooker* was based on “materials collected and arranged by Lady Hooker.” Her “careful spade work” and “active sympathy” were acknowledged in the preface, while the book was offered as a “labour of love and remembrance” by “the son of his close friend” (L. Huxley ed. 1918, vol. 1, pp. vii-viii). The editorial activity that transferred personal material to print required a combination of loyal intimacy and respectful detachment. This delicate balance was often achieved through a division of labor between a family member and a professional colleague. Though not a botanist, Leonard referred the proofs to Hooker’s successor at Kew gardens, William Thiselton-Dyer. Often, the expert who authenticated scientific achievement was also a good friend or relation–in this case, Hooker’s son-in-law. Familial bonds and friendship never compromised the truth but were essential to it.

Because science was supposed to be disinterested and transparent, the privacy of scientific lives could become highly contested. The memoir of the Scottish geologist James David Forbes was edited by three physicists who were also former colleagues and friends. The biographers noted in their preface that every statement regarding Forbes as an investigator had been read and “endorsed by Sir William Thomson, as double guarantee for accuracy, which in delicate matters of discovery, is of high value” (Shairp, Tait, and Adams-Reilly 1883, pp. ix-xi). The memoir was pounced upon by John Tyndall, an opponent of Forbes’s glacial theory. Tyndall sought to correct the biographers by reassessing Forbes’s character. He was not a man of “scrupulous and chivalrous honour” but one consumed by the pursuit of honor itself. Tyndall recounted an exchange at the British Association meeting in York in which “a purely scientific remark [on glaciers] was turned, with sudden acerbity, into a personal matter.” Tyndall insisted that his own criticism was completely impersonal, motivated only by the pursuit of truth. He concluded with a discussion of a “darkly expressed passage” in the memoir, a section in which Forbes’s biographers had noted his carefully ordered correspondence, every letter written to him preserved and docketed, every letter of his own retained in the form of a copy. Among this storehouse were accounts of private meetings in academic committees. The material, they remarked, was of great interest in revealing “that mysterious wire-pulling which seems inseparable from every transaction involving honours.” Having tipped the readers, the biographers then withheld the letters, trusting that they would be safely deposited under seal until all of the actors were dead. Tyndall was outraged and demanded the immediate publication of the letters (Tyndall 1873, pp. 23–25, 31–35). Science was a public good: its character rested on full disclosure and openness.

Unlike Tyndall (and his good friend Thomas Henry Huxley), Darwin shrank from public controversy. He preferred to air differences in private and used letters to address criticism and conduct debates. His bitter disputes with Richard Owen and St George Mivart arose when the boundaries of private and public communication were, in his eyes, disrespected and bonds of friendship undermined by ambition. Darwin occasionally marked letters “Private” to guard against their publication in newspapers. As his fame increased, he grew wary of breaches of confidence. After his death, his legacy quickly became a battleground. Even within the family, there was intense negotiation over what to make public. Who was Darwin’s *Life* for? In what ways was it exemplary? A heated debate ensued over whether his personal memoir (“Recollections of my life and character”) should be published. He had composed it for his family and had distributed handwritten copies to his children. Emma thought parts of it were hastily written and unrepresentative of her husband’s beliefs. Religion was the touchiest subject. The couple had resolved their own differences largely through respectful silence. Darwin had almost completely avoided the subject in print, but he was more expansive in his “Recollections,” even comparing religious conviction to monkeys’ instinctive fear of snakes. Darwin’s sons were keen to publish the manuscript in full, arguing that others were already making pronouncements in his name. In the end, a compromise was struck, and passages that Emma found objectionable were silently removed. The long discussion of religion was substantially shortened and moved to the end of the first volume, where it appeared with letters on the compatibility of evolution with belief in God and the insolubility of religious problems (F. Darwin ed. 1887, vol. 1, pp. 304–313; Barlow 1958). Emma was happy with the result, for she wrote to Francis shortly after publication, “I have been reading the scientific letters, and in almost every one there is some characteristic but which charms one. A little mention of me in a letter … sent me to bed with a glow about my heart coming on it unexpectedly” (Litchfield ed. 1904, vol. 2, pp. 379–380).

The modern edition of Darwin’s correspondence seems far removed from these Victorian *Lives*, where editing was an exercise of familial affection, edifying the public while safeguarding a small community of readers (Burkhardt et al. eds. 1985–2022).^1^ Would Emma have wanted strangers with only scholarly credentials pouring over personal notes and diaries? For this reason (and others), many collections remain in private hands. An enormous debt is thus owed to Darwin’s family and descendants for preserving such a vast body of material, depositing it, and then granting public access. Now editors can be custodians of the text, not personal character. Faithful transcription is the rule, not dutiful selection. Questions of privacy are banished by publishing everything. As a result, Darwin’s correspondence is a marvelous resource for domestic life, the integration of scientific work and home, the hidden stakes of public controversy, the complexities of friendship and adversity in scientific debate.

Perhaps the most significant departure from Victorian practice is the publication of both sides of correspondence. “Of letters to my father,” Francis had remarked, “I have not made much use” (F. Darwin ed. 1883, vol. 1, p. v). When the Darwin Project began, many scholarly editions still included only the letters of the celebrated author (Voltaire, Jean-Jacques Rousseau, Charles Dickens).^2^ Correspondence was considered a form of authorship, an extension of the literary or philosophical oeuvre. Editing everything to an exacting standard proved to be a prolonged process. Burkhardt thought the Darwin correspondence would take about five years to complete (!), but nearly a decade was spent on the groundwork for the first volume, collecting, transcribing, and dating some 14,000 letters. When the volumes started to appear, it was at an average rate of one per year of Darwin’s life. The editors were not exactly part of Darwin’s family, but they did spend more time with him than his children had. Many of the current team who will complete the edition in 2022 have passed some 25 years in proofreading, typesetting, researching, and annotating. So much time “up close and personal” brings a deep familiarity that is unlike the experience of biographers because of the constant immersion in detail, the relentless and fine grained chronology, day by day, month by month, with never a moment for narrative. This sense of intimacy with one’s subject sits strangely with the extreme impersonality and collective nature of the scholarship.

One of the most rewarding aspects of the work has been the “other side” of the correspondence, the diversity of lives that intersected with Darwin’s across the broadest range of topics. Unlike in Francis’s reverent *Life*, Darwin has indeed been broken into fragments, but thousands of other lives have found a place—with over 10,000 biographical entries created in the course of editing the letters. The Project began as a start-up of the Darwin industry and has been a foundation for further Darwin studies and biographies. But one of its most important and perhaps unexpected outcomes is to shift the focus away from Darwin toward other “scientists” throughout Europe and the Americas—colonial officials, travelers, settlers, farmers, gardeners, animal breeders, clergymen and crusading secularists, women writers, educationists, and suffragists—and to follow their intricate discussions of bee pollen, worm casts, and carnivorous plants, or morality, religion, and aesthetics, extended conversations over months and even years. The limitations of any edition of letters drawn from a single life will be overcome in the future. A new digital archive of correspondence across nineteenth-century collections is now in the making, Epsilon (www.epsilon.ac.uk).^3^ This new enterprise, just underway, will be both a long-term repository for the Darwin letters and a database to which more and more records on other scientific figures will be added. Ultimately, letters and the individual *Life* may be disaggregated, but also allowing new collections to be created and new characters to emerge.

## Footnotes

1 See also Darwin Correspondence Project. University of Cambridge. https://www.darwinproject.ac.uk.

2 The Newton letters were produced according to a different model, with extensive third party correspondence, other manuscript material, and lengthy technical annotations that were a running commentary on his work (Turnball, Scott, Hall, and Trilling 1959–1978).

3 Epsilon. Explore 19th c. Scientific Correspondence. Cambridge University. https://epsilon.ac.uk/.
